# Exogenous nitric oxide promotes salinity tolerance in plants: A meta-analysis

**DOI:** 10.3389/fpls.2022.957735

**Published:** 2022-11-07

**Authors:** Md. Tahjib-Ul-Arif, Xiangying Wei, Israt Jahan, Md. Hasanuzzaman, Zahid Hasan Sabuj, Faisal Zulfiqar, Jianjun Chen, Rashid Iqbal, Khondoker M. G. Dastogeer, Abdullah Al Mamun Sohag, Sadia Haque Tonny, Imran Hamid, Ibrahim Al-Ashkar, Mohsen Mirzapour, Ayman El Sabagh, Yoshiyuki Murata

**Affiliations:** ^1^ Plant Biology and Biofunctional Chemistry Lab, Department of Biochemistry and Molecular Biology, Bangladesh Agricultural University, Mymensingh, Bangladesh; ^2^ Institute of Oceanography, College of Geography and Oceanography, Minjiang University, Fuzhou, China; ^3^ Department of Biology, York University, Toronto, ON, Canada; ^4^ Department of Biotechnology, Bangladesh Agricultural University, Mymensingh, Bangladesh; ^5^ Breeding Division, Bangladesh Sugarcrop Research Institute, Pabna, Bangladesh; ^6^ Department of Horticultural Sciences, Faculty of Agriculture and Environment, The Islamia University of Bahawalpur, Bahawalpur, Pakistan; ^7^ Environmental Horticulture Department and Mid-Florida Research and Education Center, Institute of Food and Agricultural Sciences, University of Florida, Apopka, FL, United States; ^8^ Department of Agronomy, Faculty of Agriculture and Environment, The Islamia University of Bahawalpur, Bahawalpur, Pakistan; ^9^ Department of Plant Pathology, Bangladesh Agricultural University, Mymensingh, Bangladesh; ^10^ Faculty of Animal Husbandry, Bangladesh Agricultural University, Mymensingh, Bangladesh; ^11^ Department of Plant Production, College of Food and Agriculture, King Saud University, Riyadh, Saudi Arabia; ^12^ Agronomy Department, Faculty of Agriculture, Al-Azhar University, Cairo, Egypt; ^13^ Faculty of Agriculture, Department of Agricultural Biotechnology, Siirt University, Siirt, Turkey; ^14^ Department of Field Crops, Faculty of Agriculture, Siirt University, Siirt, Turkey; ^15^ Department of Agronomy, Faculty of Agriculture, Kafrelsheikh University, Kafr el-sheikh, Egypt; ^16^ Graduate School of Environmental and Life Science, Okayama University, Okayama, Japan

**Keywords:** abiotic stress, antioxidants, NO, photosynthesis, plant growth, oxidative stress, salt stress

## Abstract

Nitric oxide (NO) has received much attention since it can boost plant defense mechanisms, and plenty of studies have shown that exogenous NO improves salinity tolerance in plants. However, because of the wide range of experimental settings, it is difficult to assess the administration of optimal dosages, frequency, timing, and method of application and the overall favorable effects of NO on growth and yield improvements. Therefore, we conducted a meta-analysis to reveal the exact physiological and biochemical mechanisms and to understand the influence of plant-related or method-related factors on NO-mediated salt tolerance. Exogenous application of NO significantly influenced biomass accumulation, growth, and yield irrespective of salinity stress. According to this analysis, seed priming and foliar pre-treatment were the most effective methods of NO application to plants. Moreover, one-time and regular intervals of NO treatment were more beneficial for plant growth. The optimum concentration of NO ranges from 0.1 to 0.2 mM, and it alleviates salinity stress up to 150 mM NaCl. Furthermore, the beneficial effect of NO treatment was more pronounced as salinity stress was prolonged (>21 days). This meta-analysis showed that NO supplementation was significantly applicable at germination and seedling stages. Interestingly, exogenous NO treatment boosted plant growth most efficiently in dicots. This meta-analysis showed that exogenous NO alleviates salt-induced oxidative damage and improves plant growth and yield potential by regulating osmotic balance, mineral homeostasis, photosynthetic machinery, the metabolism of reactive oxygen species, and the antioxidant defense mechanism. Our analysis pointed out several research gaps, such as lipid metabolism regulation, reproductive stage performance, C4 plant responses, field-level yield impact, and economic profitability of farmers in response to exogenous NO, which need to be evaluated in the subsequent investigation.

## Introduction

Various biotic and abiotic factors affect the production of crops worldwide. Salinity is one of the most important abiotic factors that significantly limits agronomic field use and declines global crop production (Rahman et al., 2016; Mbarki et al., 2018; El Sabagh et al., 2021a). Soil salinization is increasing due to mismanaged irrigation practices and sea-level rise. Thus, salinity is becoming a significant threat to sustainable and resilient agriculture (Tester and Langridge, 2010; Zörb et al., 2019) because we have to grow 70% more food to feed the 9.3 billion population by 2050 (Shabala, 2013).

The toxic effect of salt impairs plant growth processes by creating physiological drought as excessive accumulations of ions reduce the soil water potential and essential mineral availability. Reduced water and nutrient uptake leads to osmotic stress, ion toxicity, and mineral imbalance in plant cells (Munns and Tester, 2008; Hanin et al., 2016; Rehman et al., 2019; Singhal et al., 2021). Salinity stress disrupts redox homeostasis and causes oxidative damage to cellular biomolecules by excessive production of reactive oxygen species (ROS) in plants (Hernández et al., 2001; Isayenkov, 2012; Monsur et al., 2020; Latef et al., 2021). Plants employ several physiological and biochemical defense mechanisms to alleviate salt-induced injury through mineral homeostasis, salt ion compartmentalization, compatible solute accumulation, antioxidant system upregulation, and phytohormonal regulation (Gupta and Huang, 2014; Fahad et al., 2015; Acosta-Motos et al., 2017; Hernández, 2019; Van Zelm et al., 2020; Ahmed et al., 2022; Hasanuzzaman, 2022). Chemical priming is considered an alternative strategy for improving abiotic stress tolerance in plants (Anwar et al., 2021). Exogenous application of signaling molecules can augment defensive responses and minimize salt-induced damage in plants (Roy et al., 2016; Tahjib-Ul-Arif et al., 2018; Tahjib-Ul-Arif et al., 2019; Dawood et al., 2021; Latef et al., 2021). In the last decade, exogenous nitric oxide (NO) has been extensively used to mitigate the adverse effects of salinity stress in different crops, and most researchers have found positive effects (Fan et al., 2013a; Ahmad et al., 2016; Siddiqui et al., 2017; Shams et al., 2019; Akram et al., 2020; Alnusairi et al., 2021; El Sabagh et al., 2021b).

Nitric oxide is a signaling molecule that positively influences plant growth and development and modulates abiotic stress tolerance in plants (Asgher et al., 2017). Accumulating evidence suggests that exogenous application of NO confers salinity tolerance in plants (Li et al., 2016; Klein et al., 2018; Sharma et al., 2020; Goyal et al., 2021). NO employs a variety of defense mechanisms to protect plants from salinity stress. In particular, it reinforces ion homeostasis and vacuolar compartmentalization, compatible solute accumulation, reactive oxygen species (ROS) metabolism, photosynthesis activity, and the antioxidant defense system in plants (Liu et al., 2013a; Manai et al., 2014; Kaya et al., 2015; Sohag et al., 2020; Alnusairi et al., 2021). Even though many research studies have been undertaken to determine the effect of exogenous NO on salt stress mitigation, it is difficult to provide an overall prescription for farmers based on these studies. Because the administration of correct doses, frequency, timing, and mode of application and overall positive benefits are so divergent, it is difficult to evaluate. Exogenous NO was applied under different growth conditions, such as greenhouses (Shi et al., 2007; Liu et al., 2013a), controlled growth chambers (Dong et al., 2015a; Tian et al., 2015; Adamu et al., 2018), and fields (Habib et al., 2016; Ali et al., 2017), in various methods, such as seed priming (Zheng et al., 2009; Hayat et al., 2012b), foliar pre-treatment (Tian et al., 2015; Adamu et al., 2018), and post-treatments (Liu et al., 2013a; Shen et al., 2018), and root medium (Fan et al., 2013a; Dong et al., 2015a), and at different frequencies, such as regular intervals (Fan et al., 2013a; Dong et al., 2015a), one time (Khan et al., 2012; Ali et al., 2017; Adamu et al., 2018), and continuous (Wu et al., 2011a; Tian et al., 2015) to mitigate salt stress. Although many studies on exogenous NO application have been published, it is unclear what the optimum NO concentrations are and how long or up to what salinity concentration could be alleviated using NO. Moreover, from the published research articles, it is impossible to understand how the plant clades, life forms, and growth stages are involved in NO-mediated salinity tolerance. As a result, a meta-analysis could be a viable alternative in determining the most effective method, concentration, and application duration and identifying potential research needs in this sector.

Meta-analysis is a systematic synthesis process that generates valuable summaries and uncovers new patterns or expands agreement among the findings of several investigations (Hedges et al., 1999; Lehmann and Rillig, 2015). Although meta-analysis has been widely used in medical science to synthesize information for making clinical decisions and policies, it continues to be used in several other disciplines, e.g., plant ecology and evolutionary biology, and has become more prevalent in recent years (Lau et al., 2013; Koricheva and Gurevitch, 2014; Gerstner et al., 2017; Gurevitch et al., 2018). A meta-analysis is more than just a systematic review; it also weighs the impact of an experimental treatment compared to a control group. The inference drawn from a meta‐analysis could be used in developing agricultural management practices that would otherwise be impossible from the individual, typically short-term research projects, most of which are limited to particular climatic conditions (Eagle et al., 2017). We did not come across any reports that used meta-analysis to focus on the effects of exogenous application of NO on salt tolerance. Many reports show that a particular NO level reduces salt stress in some crops. Still, a generalized recommendation for field-level applications cannot be given unless the combined outcome and underlying factors are understood. To this end, we gathered data from 62 relevant research studies found through our literature search. We assessed the effect of NO on diverse agricultural plants in response to morphological, physiological, and biochemical alterations caused by salinity stress. The current study aimed to answer the following key questions:

- What is the overall strength or magnitude of the effect of NO application in mitigating plant salt stress across various contexts?
- How much, or up to what level, can NO alleviate salinity stress, and what concentration of NO is effective for that salinity level?
- What are the roles of various factors (plant factors, NO factors, and environmental factors) in No mediated plant salinity tolerance?
- How does exogenous NO influence plant physiological and biochemical parameters?

## Materials and methods

### Literature search and selection criteria

**Figure 1:**
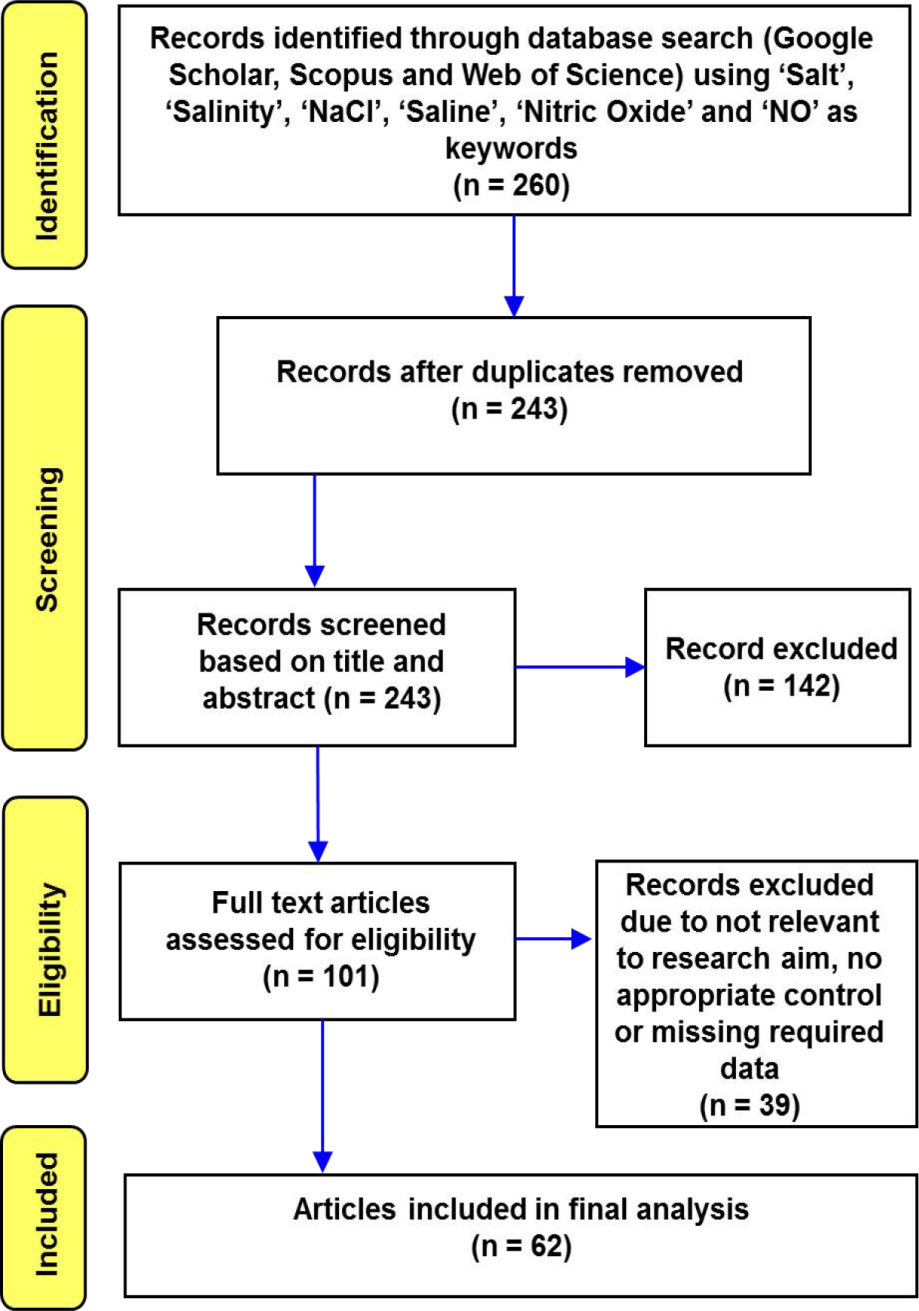
Flow chart depicting the Preferred Reporting Items for Systematic Reviews and Meta-Analysis (PRISMA) search strategy used to find and choose published literature for this analysis.

The data for this meta-analysis was gathered according to the general guidelines by Field and Gillett (2010). We used the Web of Science, Scopus, and Google Scholar databases to conduct an extensive literature search until June 2020. Our keywords were “nitric oxide AND salt stress/or salinity” and “NO AND salinity/or salt stress.” Based on the titles and abstracts of all search results, 260 articles were deemed to contain relevant information (**Figure 1**). For data collection, the articles were selected using the following set of criteria: (i) the experiment had to manipulate at least one concentration of exogenous NO, (ii) the exogenous NO application was used singly, and we avoided mixed or combined exogenous treatments in this analysis, (iii) both NO-treated and non-NO-treated plants were grown under saline and non-saline conditions, (iv) any selected parameter was investigated, and (v) the findings reported sample size, means, standard deviations/errors, or other relevant statistical information such that the outcome could be converted to a standardized measure of effect size.

Based on these criteria, we finally selected 62 studies out of 260 for analysis. The level of fertilizer applied, the growing settings (greenhouse, growth chamber, or field), the duration of time before stress was exposed, and the growth media used in our meta-analysis were all allowed to vary. The papers covered 20 years (2000–2020) and were written in English. The detailed paper selection procedure has been provided in **Figure 1** according to PRISMA guidelines.

### Data extraction

We retrieved data on dry biomass of plants, NO application, photosynthetic and enzyme parameters, and other relevant data from the studied articles. Mean values, sample sizes (replications), and standard deviations (SDs) were recorded from each investigation. We converted the presented standard errors (SEs) to standard deviations with the equation SD = SE × √ (sample size). The reported 95% CIs (confidence intervals) were translated to SDs where applicable. We employed WebPlotDigitizer V4.2 (https://automeris.io/WebPlotDigitizer/) to digitize the values of figures. Multiple treatments or combinations of plant species/cultivars from the same experiment were treated as different studies and included in the analysis as independent data units. By presuming that the studies are independent, extracting multiple studies from a single experiment may increase the reliability of that study (Gurevitch and Hedges, 1999). We estimated the mean effect size of the dataset using only a single random observation from each research paper (reduced dataset) and compared this with the effect size calculated using the entire dataset (full dataset) to investigate potential publishing biases due to non-independence from numerous observations (He and Dijkstra, 2014). We used Welch’s t-test to assess effect sizes (full dataset vs. reduced dataset) to see if data reduction might significantly impact effect size. We found no significant discrepancies because of data reduction, indicating that overrepresentation was unlikely in this study. Considering multiple observations from the same investigation to be independent is expected to improve meta-analysis statistical power (Lajeunesse and Forbes, 2003). This method has been employed in many biological meta-studies (Veresoglou et al., 2012; Mayerhofer et al., 2013; Mcgrath and Lobell, 2013; Eziz et al., 2017; Dastogeer, 2018; Dastogeer et al., 2020).

### Statistical procedure for meta-analysis

The meta-analyses were conducted in R version 4.2.1 using the “meta” (Balduzzi et al., 2019). The standardized mean difference (SMD) was measured using Hedge’s g statistic to determine the effect size for the difference between means using the “metacont” function. Hedge’s g is a meta-analysis statistic that reflects the difference in means in units of the pooled standard deviation and is favored over other measures like the log-response ratio because it has a lower Type I error rate (Lajeunesse and Forbes, 2003; Van Kleunen et al., 2010; Xie et al., 2018). In the case of our study, the SMD is recommended for meta-analyses, including studies reporting continuous outcomes (Faraone, 2008). A SMDs = 0 indicates that the two treatments (NO-treated or non-treated) have a similar effect, whereas a SMDs >0 reflects how the NO-exposed samples surpassed the non-exposed samples and vice versa. In particular, SMD values of 0.3, 0.5, and 0.8 imply low, moderate, and high impact sizes, respectively (Cohen, 1988). The overall effect was calculated using a random-effects model. A random-effects model was used because diverse types of experiments were included in the model. Due to the diverse locations, conditions, experimental settings, and methodologies utilized in the individual investigations, it is unlikely that all of them would predict a similar effect size (Borenstein et al., 2009). We computed 95% confidence intervals (CIs) and interpreted them in such a way that when the 95% CIs exclude zero, the effect size (SMD) is assumed to be significant. The Sidik–Jonkman estimator (Sidik and Jonkman, 2005) was used with the Hartung–Knapp adjustment (HKSJ) to estimate the random effects variance. When the combined studies are of varying sizes and demonstrate between-study heterogeneity, HKSJ creates inflated error rates, but it outperforms the widely used DerSimonian and Laird technique approach (Sidik and Jonkman, 2007; IntHout et al., 2014). Higgin’s I^2^ and Cochran’s Q statistics were employed to measure and test for statistical heterogeneity. I^2^ is the ratio of actual heterogeneity to overall heterogeneity across reported effect sizes, whereas Q is the weighted deviation from the summary effect size attributable to heterogeneity other than due to the sampling error (Higgins and Thompson, 2002; Higgins et al., 2003; Huedo-Medina et al., 2006). In general, I^2^ values vary from 0% to 100%, and the values of <25, 25–75, and >75% indicate small, medium, and high heterogeneity, respectively (Higgins et al., 2003).

### Finding publication biases and adjustment

We used several methods to test the publication bias for each dataset. We visually examined asymmetry in funnel plots, employed “trim-and-fill” analysis, and ran Begg and Mazumdar rank correlation tests based on Kendall’s tau, the Egger regression test, and p-curve analysis (Begg and Mazumdar, 1994; Egger et al., 1997; Simonsohn et al., 2014). If these tests revealed significant bias, we used the trim-and-fill procedure to correct the biases and calculate the effect sizes (SMD), CIs, and heterogeneity statistics (Schmidt and Hunter, 2015) (Duval and Tweedie, 2000). We constructed subgroups from the studies based on the moderator subgroups. We applied trim-and-fill to the subgroups if any subgroups had biases identified by the above tests (Schmidt and Hunter, 2015).

### Selected subgroup analyses

We performed subgroup analyses on the data to investigate the influence of factors, such as plant identity, NO treatment settings, plant life cycle, and salinity duration on the shoot and root dry biomass parameters. Using the “dmetar” package in R, we conducted a mixed-effects model with the subgroups as the fixed-effects element (Harrer et al., 2019). The overall impact size for each subgroup was determined using a random-effects model, and then we used a fixed-effects model to test between-subgroup differences (Borenstein and Higgins, 2013).

This approach is appropriate when the subgroup levels under examination are expected to be exhaustive for the characteristics and are not picked at random. Because most of the subgroups in our analysis were fixed, such as plant life cycle (annual and perennial) and plant clade (monocot or dicot), we hypothesized that a mixed-effects model would be a good fit. A factor needs to be reported in at least five studies across two separate papers to be included in the analysis as a subgroup variable. Initially, we considered different salinity levels (low, moderate, and high) but found no significant effects of salinity levels on the shoot and root biomass production (**Table S2**). Thus, in this meta-analysis, we consider only saline and non-saline conditions.

*Growth condition*: Plant growth conditions were categorized into three groups: greenhouse, growth chamber, and field.

*Method of NO application*: the methods of NO were divided into four categories: seed priming, root medium, foliar pre-treatment, and foliar post-treatment.

*Duration of NO application*: The period of NO supplementation was divided into three categories: regular interval, one time, and continuous.

*Duration of salinity*: The period of salinity treatment was divided into three categories: short (<8 days), moderate (8–21 days), and long (>21 days).

*Plant growth stages were divided into two groups*: seedling and germination

*Plant clades were classified into two groups*: monocots and dicots

*Plant life forms were classified into three classes*: vines, graminoids, and forbes

## Results

**Figure 2:**
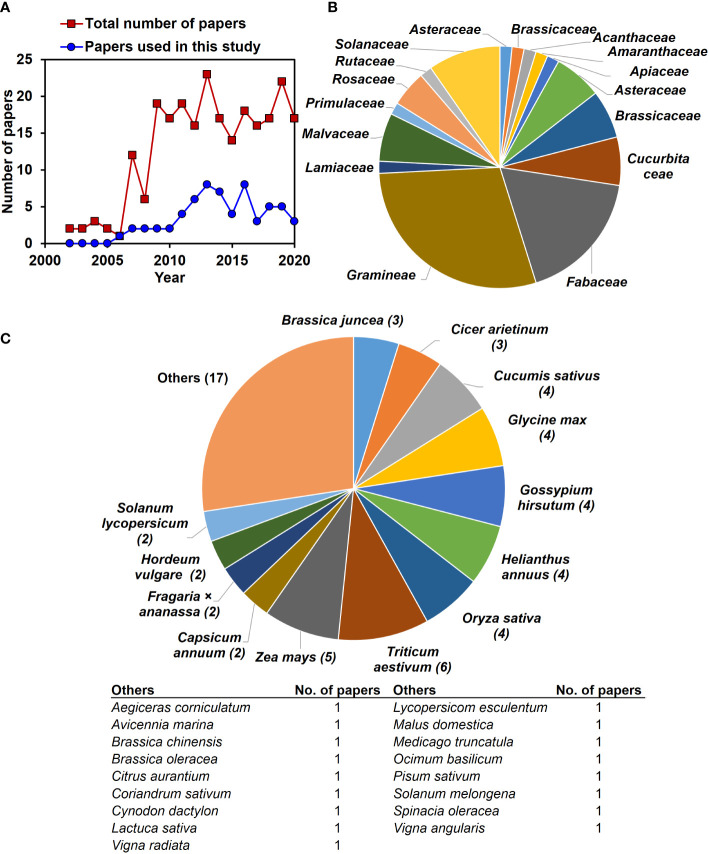
The total number of published articles, plant families and species about exogenous NO effects on plant stress physiology for salt tolerance available in the ‘Web of Science’ and ‘Scopus’ databases between 2000 and 2020, **(A)** the inlets in the main plots display the total number of articles found in the search, while the main plots exhibit the number of publications by year. **(B)** The pie chart represents the sixteen diverse families of the studied plants. **(C)** The pie chart shows the 30 diverse plant species from 16 families across the used articles in this study.

In this study, data were collected from 2000–2020, and after 2005, more papers were published on exogenous NO-mediated salinity stress mitigation (**Figure 2A**). We investigated the effects of exogenous application of NO on 32 plant response parameters at various levels of salt stress. In this study, the summary effect sizes for non-stressed plants were also examined for comparison. Plants were represented by 30 species from 16 families across the 62 articles (**Figures 2B, C**). *Triticum aestivum* (6), *Zea mays* (5), *Oryza sativa* (4), *Helianthus annuus* (4), *Gossypium hirsutum* (4), *Glycine max* (4), and *Cucumis sativus* (4) were the most commonly studied plant species (**Figure 2C**).

### Effects of exogenous NO on plant growth parameters and yield under salinity

**Figure 3:**
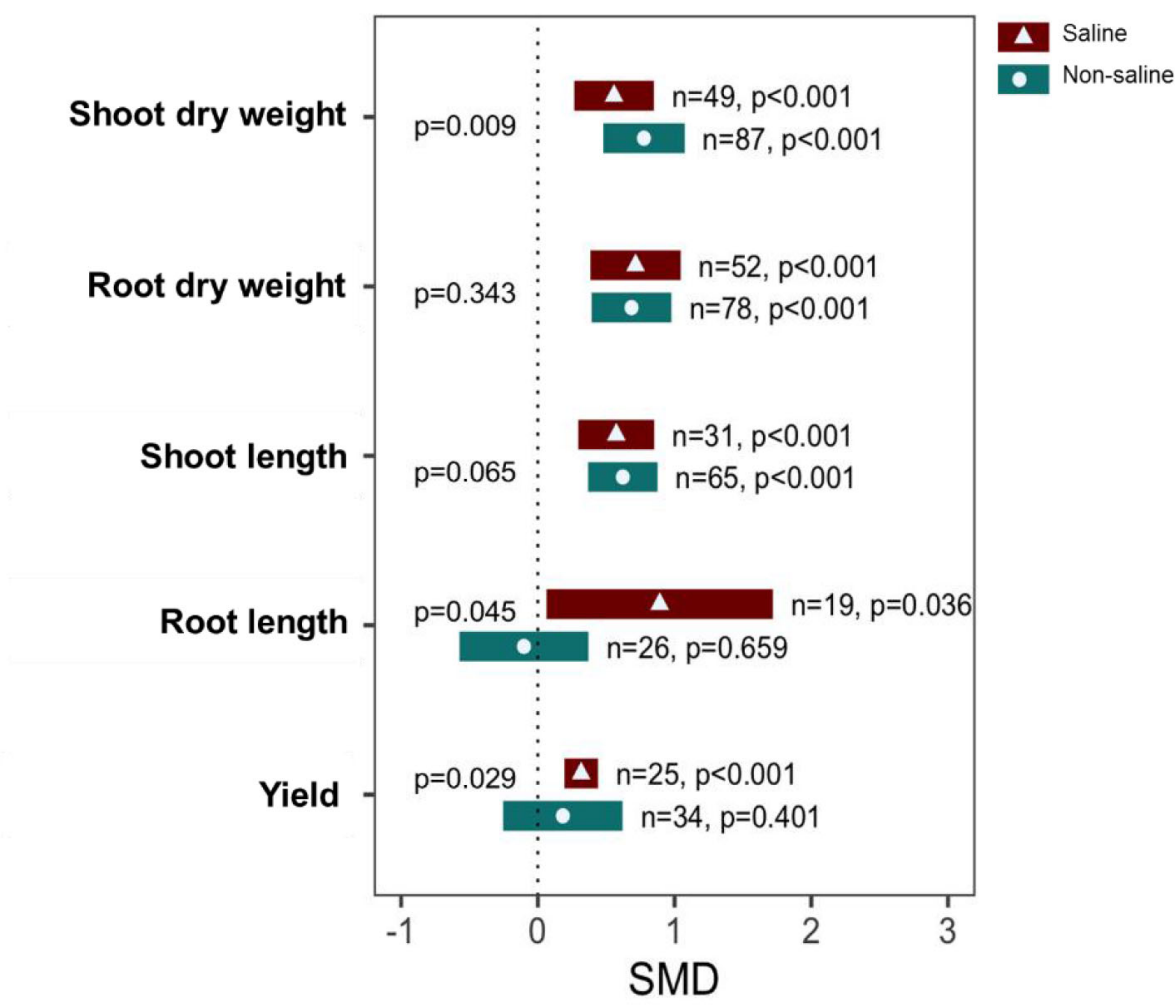
Growth responses and yield of exogenous NO-treated plants compared with those of non-NO-treated plants under non-saline and saline conditions. Error bars are effect size (SMD) means ±95% CIs. Where the CIs do not overlap the vertical dashed lines, the effect size for a parameter is significant, i.e., the growth responses of NO-treated plants were significantly different from those of non-NO-treated plants. *n*, the number of studies included in the meta-analysis; *p*, the significance level of SMD.

Exogenous NO application significantly increased SDW, RDW, and SL both under saline (*p <*0.001) and non-saline (*p <*0.001) conditions (**Figure 3**). The subgroup analysis indicated that there is a significant difference in the effect size of SDW between saline and non-saline conditions. However, the effect sizes of RDW and SL between saline and non-saline conditions were statistically similar because of the overlapping confidence interval values (**Figure 3**). Moreover, exogenous application of NO significantly increased RL (*p* = 0.036) and yield (*p <*0.001) under saline conditions, although no significant effects were observed under non-saline conditions (**Figure 3**). There are also significant differences in the effect sizes of RL (*p* = 0.045) and yield (*p* = 0.029) between salt-stressed and non-stressed conditions (**Figure 3**; **Supplementary Table 3**).

### Categorical analysis of the effects of exogenous NO on SDW

**Figure 4:**
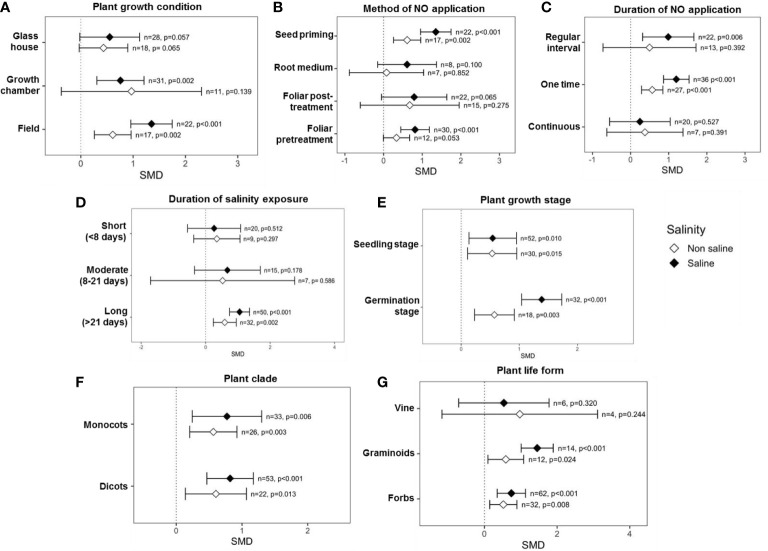
Effects of exogenous NO on SDW under saline and non-saline conditions for various categorical variables such as **(A)** growth conditions, **(B)** method of NO application, **(C)** duration of NO application, **(D)** duration of salinity, **(E)** plant growth stages, **(F)** plant clade, and **(G)** plant life form. The error bars are the effect size means ±95% CIs. Where the CIs do not overlap the vertical dashed lines, the effect size for a parameter is significant, i.e., the growth responses of NO-treated plants were significantly different from those of non-NO-treated plants. *n*, the number of studies included in the meta-analysis; *p*, the significance level of SMD.

Categorical variables considered in the analysis showed that multiple factors influence the impact of exogenous NO on SDW, e.g., plant growth condition, method, time, and duration of NO application, plant factors, and salinity stress factors. For example, experiments that were performed under growth chamber (*p* = 0.002) and field conditions (*p <*0.001) showed significant effects on SDW under saline conditions (**Figure 4A**). Among the methods of exogenous NO application, “seed priming” (*p <*0.001) and “foliar application” (*p <*0.001) before exposure to salinity had a significant effect on the SDW production under salinity stress. On the other hand, “root medium” and “foliar post-treatment” showed a non-significant effect on SDW production both under saline and non-saline conditions (**Figure 4B**). The effect sizes (SMDs) of “root medium” with NO application were based on only a few studies (*n* = 8, saline and *n* = 7, non-saline), and the CIs interval is very long. Moreover, the CIs interval in the case of “foliar post-treatment” NO application is very long (**Figure 4B**). Thus, further studies should be performed focusing on “root medium” and “foliar post-treatment” with NO application. Applications of NO for “one time” (*p <*0.001) and “regular interval” (*p* = 0.006) significantly improved the SDW under saline conditions, but “continuous” application was not effective both under saline and non-saline conditions (**Figure 4C**). However, the CIs interval for ‘continuous’ NO treatment was very long, which suggests variable results among different studies (**Figure 4C**). Exogenous NO application most effectively improved SDW under ‘long (>21 days)’ duration salinity but did not improve under ‘short (<8 days)’ and ‘moderate (8-21 days)’ duration salinity stress (**Figure 4D**). Surprisingly, the effect of NO on SDW became stronger as the duration of salt treatment increased (**Figure 4D**). The application of NO significantly improved SDW production under saline conditions at both the seedling (*p* = 0.010) and germination (*p <*0.001) stages, where NO-mediated SDW improvement was more pronounced at the germination stage (**Figure 4E**). In both dicot and monocot plants, application of NO increased SDW more than non-NO treatment under salinity and non-salinity conditions (**Figure 4F**). As is evident in the figure (**Figure 4G**), application of NO on forbes and graminoid plants tended to increase SDW production under both saline and non-saline conditions.

### Categorical analysis of the effects of exogenous NO on RDW

**Figure 5:**
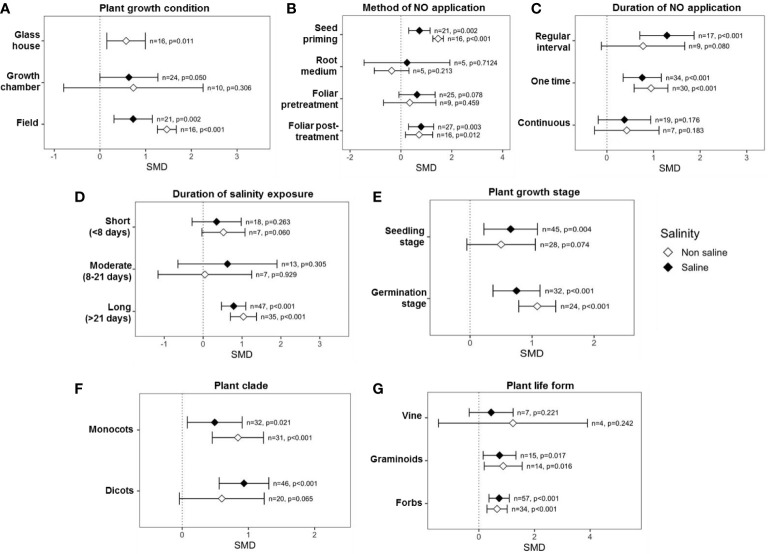
Effects of exogenous NO on RDW under saline and non-saline conditions for various categorical variables such as **(A)** growth conditions, **(B)** method of NO application, **(C)** duration of NO application, **(D)** duration of salinity, **(E)** plant growth stages, **(F)** plant clade, and **(G)** plant life form. The error bars are the effect size means ± 95% CIs. Where the CIs do not overlap the vertical dashed lines, the effect size for a parameter is significant, i.e., the growth responses of NO-treated plants were significantly different from those of non-NO-treated plants. *n*, the number of studies included in the meta-analysis; *p*, the significance level of SMD.

According to categorical variables evaluated in the analysis, the effect of exogenous NO on RDW is influenced by numerous parameters, including plant growth settings, method, time, and duration of NO administration, plant factors, and salt stress factors. For example, experiments that were undertaken at field conditions (*p* = 0.002) displayed significant impacts on RDW under saline conditions (**Figure 5A**). Among the methods of exogenous NO application, “seed priming” (*p* = 0.001) and “foliar post-treatment” (*p* = 0.003) had a significant effect on the RDW production under the absence and presence of salinity. On the contrary, “root medium” and “foliar application” exhibited no significant effect on RDW production in the case of both salinity and non-salinity conditions (**Figure 5B**). The effect sizes (SMDs) of the “root medium” NO application were based on only a few studies (*n* = 5, saline and *n* = 5, non-saline), and the CIs interval is very long under saline conditions (**Figure 5B**).

NO application for “one time” (*p <*0.001) and “regular interval” (*p <*0.001) considerably enhanced RDW in a saline environment, while “continuous” application was ineffective in both saline and non-saline conditions (**Figure 5C**). However, the CIs for “continuous” NO treatment were extremely wide, implying that findings differed greatly between investigations (**Figure 5C**). Exogenous NO treatment enhanced RDW the most under “long (>21 days)” (*p <*0.001) duration salinity, while no significant improvement was found under “short (0–8 days)” and “moderate (9–21 days)” duration salt stress (**Figure 5D**). Interestingly, as the duration of salt treatment increased, the effect of NO on RDW grew stronger (**Figure 5D**). Plant growth stage had no effect on RDW generation; plants acquired considerably greater shoot biomass in response to exogenous NO treatment than non-NO treatment at both seedling (*p <*0.001) and germination stages (*p <*0.001) (**Figure 5E**). Under saline and non-saline circumstances, the application of NO raised SDW in both dicot and monocot plants more than non-NO treatment (**Figure 5F**). As is evident in the figure (**Figure 5G**), NO treatment on forb and graminoid plants is supposed to enhance RDW generation under salinity and non-salinity conditions.

### Effects of exogenous NO on plant photosynthetic attributes

**Figure 6:**
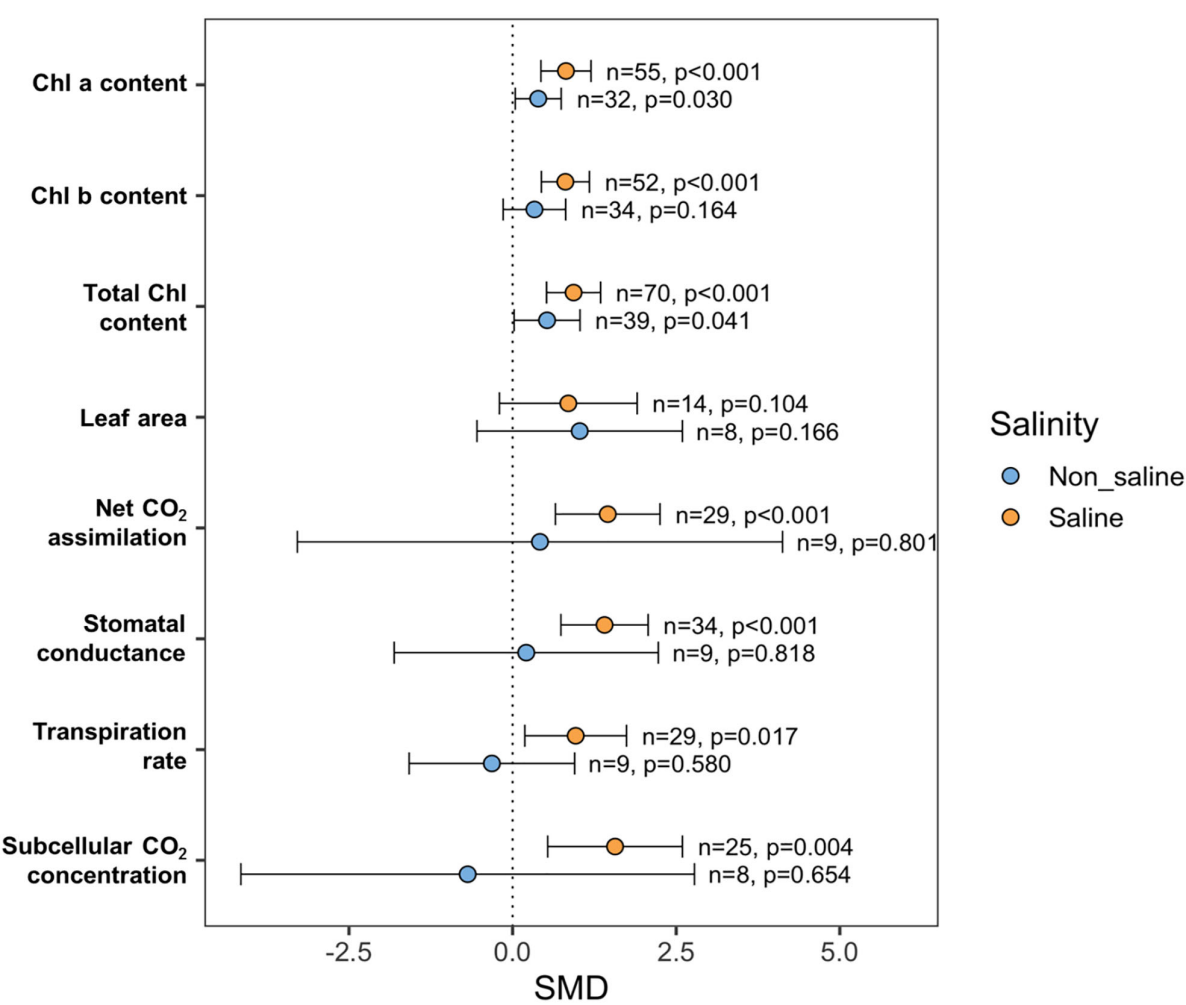
Effects of exogenous NO on photosynthesis-related parameters such as chlorophyll-a (Chla), chlorophyll b (Chlb), total chlorophyll (total Chl) content, leaf area (LA), net CO_2_ assimilation (Pn), stomatal conductance (Gs), transpiration (E) and sub-cellular CO_2_ concentration (Ci) under saline and non-saline conditions. The error bars are the effect size means ±95% CIs. Where the CIs do not overlap the vertical dashed lines, the effect size for a parameter is significant, i.e., the growth responses of NO-treated plants were significantly different from those of non-NO-treated plants. *n*, the number of studies included in the meta-analysis; *p*, the significance level of SMD.

Most of the photosynthetic parameters, such as chlorophyll a (Chla), chlorophyll b (Chlb), total Chl, rate of photosynthesis (Pn), stomatal conductance (Gs), transpiration (E), and subcellular CO_2_ concentration (Ci), were significantly influenced by exogenous NO application under saline and non-saline conditions (**Figure 6**). The subgroup analyses revealed that the favorable effects of exogenous NO treatment on particular plant photosynthetic indices, including Chla, Chlb, Pn, Gs, E, and Ci, were higher when plants were exposed to salt stress than in non-stressed plants (**Figure 6**). For example, the Chla content was significantly influenced by exogenous NO application under saline (*p <*0.001) and non-saline (*p* = 0.03) conditions (**Figure 6**). Exogenous NO administration significantly impacted Chlb content in saline (*p <*0.001) but not in non-saline (p = 0.164) conditions (**Figure 6**). There is no effect of exogenous NO application on leaf area (LA) both under stressed and non-stressed conditions (**Figure 6**). The Pn, Gs, E, and Ci were significantly improved by exogenous NO application under saline conditions (*p <*0.001, Pn, Gs; *p* = 0.017, E; *p* = 0.004, Ci) but not under non-saline conditions (**Figure 6**). However, all of the measures studied tended to be more variable under non-stress conditions compared to saline-stress environments, as evidenced by their higher confidence interval values (**Figure 6**).

### Effects of exogenous NO on plant water relations and nutrient homeostasis

**Figure 7:**
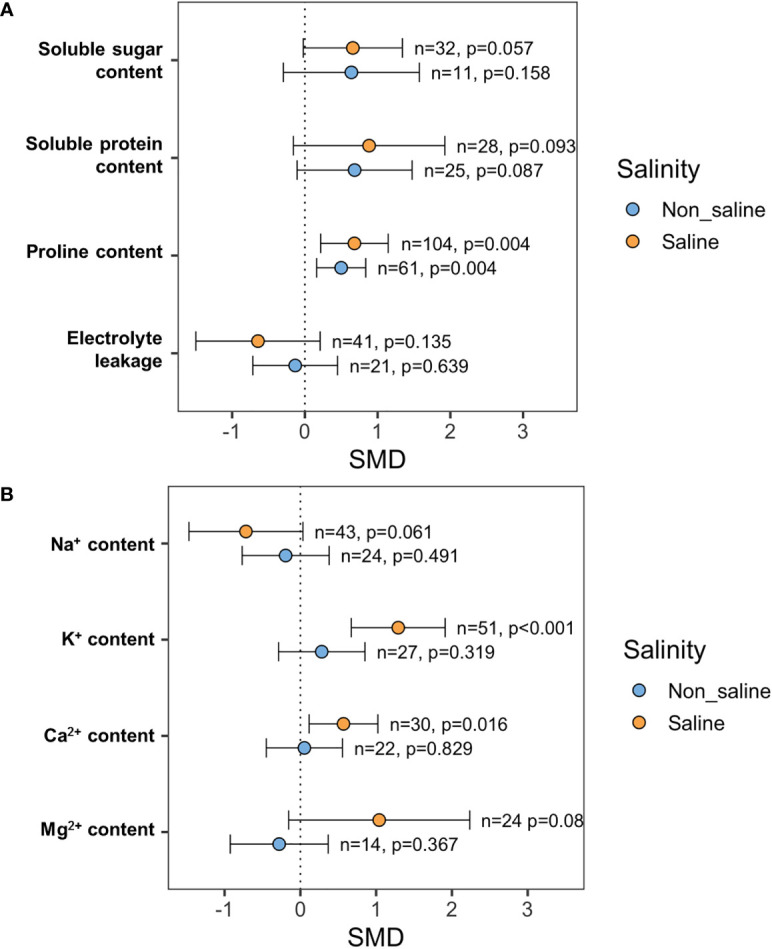
Effects of exogenous NO on **(A)** soluble sugars (SS), soluble proteins (SP), proline content, and electrolyte leakage (EL) and **(B)** Na^+^, K^+^, Ca^2+^, and Mg^2+^ contents under saline and non-saline conditions. The error bars are the effect size means ±95% CIs. Where the CIs do not overlap the vertical dashed lines, the effect size for a parameter is significant, i.e., the growth responses of NO-treated plants were significantly different from those of non-NO-treated plants. *n*, the number of studies included in the meta-analysis; *p*, the significance level of SMD.

Exogenous NO application did not increase SS and SP content significantly both under stressed (*p* = 0.057, SS; *p* = 0.093, SP) and non-stressed (*p* = 0.158, SS; *p* = 0.087, SP) conditions (**Figure 7**). However, exogenous application of NO improved Pro content significantly both under saline (*p* = 0.004) and non-saline (*p* = 0.004) conditions (**Figure 7**). Electrolyte leakage (EL) decreased in response to exogenous NO application under salt stress conditions (*p* = 0.135), but this change is non-significant because of the higher CI interval (**Figure 7**).

The leaf K^+^ content (*p <*0.001) and Ca^2+^ content (*p* = 0.016) in NO-treated plants were consistently higher than those in non-NO-treated plants under salinity conditions (**Figure 7**). Under saline conditions, leaf Na^+^ content was dropped in response to exogenous NO delivery, but this shift was non-significant (*p* = 0.061) due to higher CI intervals (**Figure 7**). Moreover, leaf Mg^2+^ content in the salt-stressed plants was increased in reaction to the administration of exogenous NO. However, this change is non-significant (*p* = 0.08) because of greater CI intervals (**Figure 7**).

### Effects of exogenous NO on plant oxidative damage and antioxidant systems

**Figure 8:**
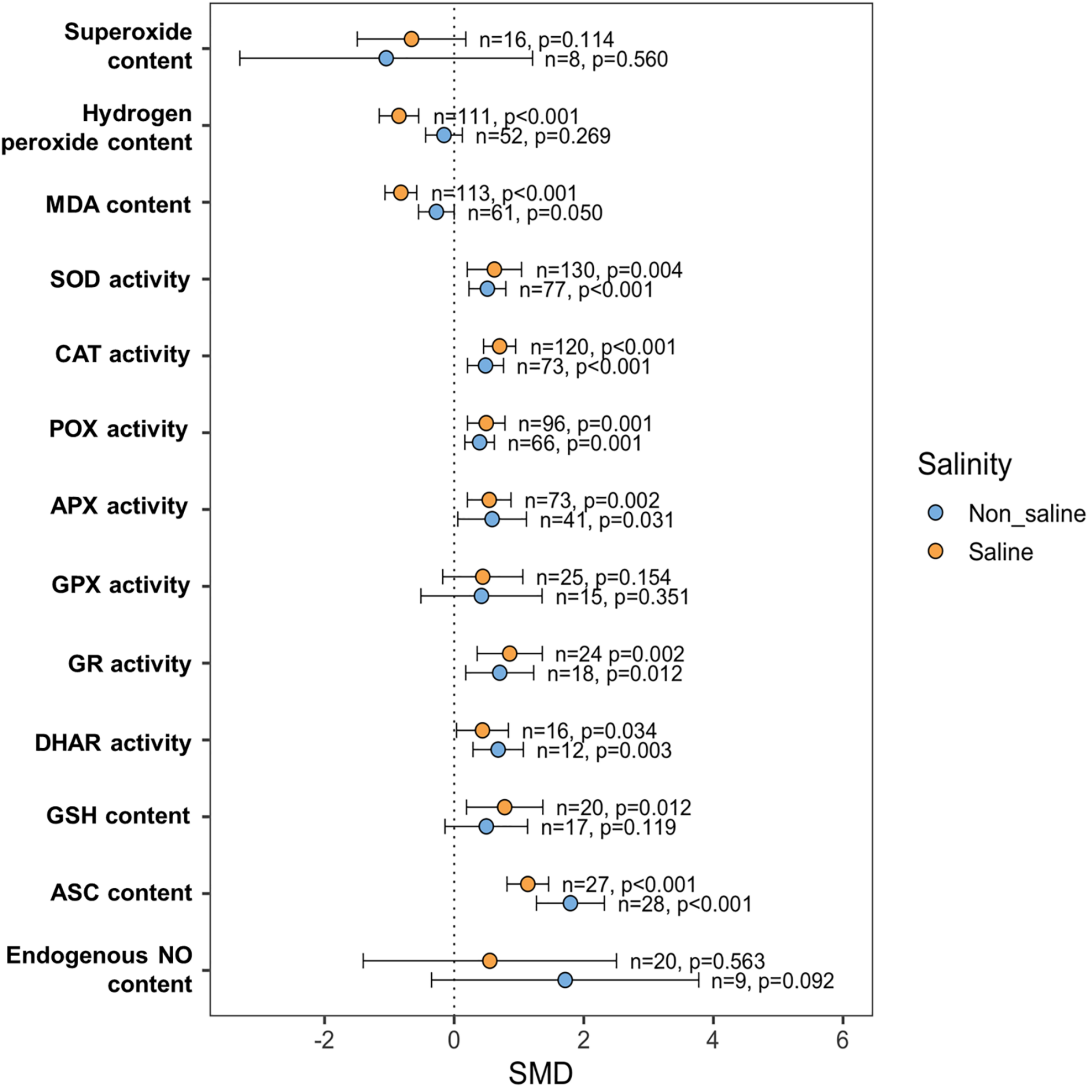
Exogenous NO effects on ROS scavenging and antioxidant capacity such as superoxide, hydrogen peroxide (H_2_O_2_), malondialdehyde (MDA), superoxide dismutase (SOD), catalase (CAT), peroxidase (POX), ascorbate peroxidase (APX), glutathione peroxidase (GPX), glutathione reductase (GR), dehydroascorbate reductase (DHAR), glutathione (GSH), ascorbate (ASC) and endogenous NO content in salt-induced plants. The error bars are the effect size means ±95% CIs. Where the CIs do not overlap the vertical dashed lines, the effect size for a parameter is significant, i.e., the growth responses of NO-treated plants were significantly different from those of non-NO-treated plants. *n*, the number of studies included in the meta-analysis; *p*, the significance level of SMD.

In non-stressed conditions, there was no change in H_2_O_2_ and MDA buildup between NO-treated and non-NO-treated plants (**Figure 8**). However, exogenous NO-treated plants had significantly lower MDA levels (*p <*0.001) and H_2_O_2_ content (*p <*0.001) than non-NO-treated plants under saline conditions (**Figure 8**). The effect of exogenous NO on superoxide content reduction was not substantial both under saline (*p* = 0.114) and non-saline (*p* = 0.560) conditions, though the NO treatment reduced the superoxide content under saline conditions (**Figure 8**). Both under saline and non-saline conditions, NO-treatment increased SOD (*p* = 0.004 and *p <*0.001, respectively), CAT (*p <*0.001 and *p <*0.001, respectively), POX (*p* = 0.001 and *p* = 0.001, respectively), APX (*p* = 0.002 and *p* = 0.031, respectively), GR (*p* = 0.002 and *p* = 0.012, respectively), and DHAR (*p* = 0.034 and *p* = 0.003, respectively) activity than non-NO-treatment (**Figure 8**). However, in both saline and non-saline environments, there was no difference in GPX activity between NO-treated and non-NO-treated plants (**Figure 8**). Among the non-enzymatic antioxidants, GSH content was significantly increased only under saline (*p* = 0.012) conditions, but ASC content was increased both under saline and non-saline conditions in response foliar NO application than non-NO application (**Figure 8**). The endogenous NO content did not change due to the application of exogenous NO in comparison to non-NO treatment both under saline and non-saline conditions (**Figure 8**).

## Discussion

It is urgent to find an economical and effective technique to reduce the damage caused by salinity stress in crops because it causes significant yield loss. Exogenous administration of some signaling molecules has a vast potential for reducing the negative consequences of abiotic stressors. In this regard, exogenous NO has been intensively studied in the last 20 years and can alleviate the harmful effects of salt stress in various plant species. In general, most research studies found that exogenous application of NO enhances salinity stress tolerance in plants, implying that it improves growth or yield under salinity stress compared to the salinity stress condition alone. However, several questions remain: at what concentration, which method, or how long did NO application show the best growth performance or yield? Does exogenous NO enhance the yield of crops? Do plant species or growth stages and conditions affect the NO-mediated effects on plants? Which physiological or biochemical processes are most modulated by exogenous NO application? A meta-analysis was performed to find the answers to those questions.

### Does exogenous NO improve plant phenotypic traits and yield during salinity stress?

Salt stress negatively impacts growth parameters (e.g., shoot–root length and root–shoot dry weight) and yield production due to increased toxicity, osmotic effect, and oxidative stress (Yildirim et al., 2009; Shams et al., 2016; Ahmad et al., 2018). The meta-analysis revealed that exogenous NO substantially affects some physiological and biochemical attributes, particularly plant growth and development, photosynthesis, and defense mechanisms to repair oxidative injury under salt stress. Exogenous NO application in different conditions displays divergence from the corresponding diagram. The effects of different salinity regimes on plant growth regulation, such as shoot and root dry weight, shoot–root growth, and comparable output, were observed in several crops, such as wheat (*Triticum aestivum*) (Perveen et al., 2011; Perveen et al., 2012), rice (*Oryza sativa*) (Iqbal et al., 2015), sorghum (*Sorghum bicolor*) (Bashir et al., 2011), and pearl millet (*Pennisetum glaucum*) (Hussain et al., 2008). According to our findings, NO treatment facilitates growth and yield under salinity-induced circumstances, where NO, as a signaling agent, works as a stress protectant (Parankusam et al., 2017). Several prior studies in maize (*Zea mays*), rice, wheat, tomato (*Lycopersicon esculentum*), and marigold (*Calendula officinalis*) showed an increase in shoot–root length and shoot–root dry weight (Farooq et al., 2009; Molassiotis et al., 2010; Bai et al., 2011; Wu et al., 2011a; Jabbarzadeh et al., 2018; Sehar et al., 2019). Our meta-analysis showed that exogenous NO confers plant salinity tolerance by reestablishing mineral uptake, osmolyte accumulation, and antioxidant enzyme activity, ensuring incredible plant growth and yield (**Figures 7**, **8**).

Salinity induces water scarcity in the root zone and immediately affects plant water status in a short period. On the other hand, the plant recovers over several hours and returns to a modest, steady growth rate. The second phase, which increases over time, is driven by the toxicity of excess Na^+^ and Cl^−^ ions that concentrate in the cytoplasm. Furthermore, plants under salinity stress require extra energy to counteract the harmful effects of Na^+^ ions, and they are also vulnerable to nutritional deficiency. These processes harm plant growth and biomass production (Munns and Tester, 2008; Isayenkov and Maathuis, 2019; Tabassum et al., 2021). Our analysis confirmed that exogenous NO supplementation significantly improved the SDW and RDW in both salt-stressed and non-stressed plants (**Figure 3**). Exogenous NO might improve shoot and root biomass production by enhancing photosynthetic pigment, nutrient uptake, and antioxidant enzymatic activity and mitigating oxidative damage in salt-stressed plants, including rice (Mostofa et al., 2015), maize (Kaya et al., 2015), wheat (Kausar et al., 2013), chickpea (*Cicer arietinum*) (Ahmad et al., 2016), tomato (Wu et al., 2011a), and pepper (Capsicum *annum*) (Shams et al., 2019).

### How do different sub-categories affect NO-mediated salinity stress tolerance in plants?

This meta-analysis showed that the effects of exogenous NO on plant biomass yield were context-dependent, with various elements playing key roles. For example, exogenous NO significantly increased SDW in field and growth chamber studies, whereas RDW was significantly improved only in field environments under salt stress (**Figures 4A**, **5A**). Plants were typically grown in plots in greenhouses or growth chambers (Egbichi et al., 2014; Jamali et al., 2015; Siddiqui et al., 2017; Khoshbakht et al., 2018; Shams et al., 2019; Jabeen et al., 2021), and many researchers failed to observe changes in root biomass in response to NO under salinity conditions, possibly because optimal root growth is reduced under these growth conditions, as supported by our meta-analysis (**Figures 4A**, **5A**). Thus, studies in the field are required to understand root phenomics in response to NO under salt stress. It is worth mentioning that all the field-level experiments were conducted at the germination stage (Habib et al., 2016; Ali et al., 2017).

This meta-analysis showed that seed priming and foliar pre-treatment were the most effective methods for NO application to plants (**Figure 4B**). The exogenous NO applications before exposure to salt stress might boost the metabolic activity, energy production, and stress tolerance mechanisms of the plant, improving plant growth responses in response to salinity. On the contrary, root medium showed no noticeable impact on shoot and root biomass accumulation under saline conditions (**Figures 4B**, **5B**). However, this proposition was based on only a few investigations; therefore, further studies should be performed on these NO application methods. NO application as seed priming and foliar post-treatment markedly increased root dry biomass under salt stress (**Figure 5B**). As plant roots are directly in contact with Na^+^ ions during salinity stress, foliar post-treatment might activate the NO-based defense machinery at first in roots to trigger a salinity tolerance mechanism. NO triggers root tip elongation and lateral adventitious root development (Correa-Aragunde et al., 2004) and integrates the ABA–IAA signaling network of root system responses in tomatoes under salt stress (Santos et al., 2020). As seed priming with NO showed the maximum effect on root growth improvement (**Figure 5B**), this method could be used for successful early seedling establishment in salinity soils. Interestingly, foliar pre-treatment and “root medium” showed no considerable effect on root dry mass production under salinity conditions (**Figure 5B**). However, a few papers on root medium-based studies have been found and examined, and more research into these NO-based approaches is needed.

Both one time and regular interval treatments of exogenous NO were efficient for the significant shoot and root dry biomass production in the presence of salinity (**Figures 4C**, **5C**). These findings are in agreement with the results on maize (Kaya et al., 2015), chickpea (Ahmad et al., 2016), broccoli (*Brassica oleracea*) (Akram et al., 2020), and chicory (*Cichorium intybus*) (Abedi et al., 2021) in the case of the regular interval, while in maize (Keyster et al., 2012), cotton (Liu et al., 2013a), and mustard (*Brassica Juncea*) (Khator and Shekhawat, 2020) for the one-time treatment. However, “continuous” NO treatment limited shoot and root growth under salinity (**Figures 4C**, **5C**), suggesting that a continuous supply of exogenous NO could be toxic to plants instead of providing cellular antioxidant protection. The interaction of NO with ROS accounts for direct sources of both toxicity and protection (Beligni and Lamattina, 1999). ROS acts as a signal for the activation of defense responses when NO is present in small concentrations (Dangl et al., 1996). Higher levels of NO created by unregulated ROS formation, on the other hand, inflict serious damage to plants (Beligni and Lamattina, 1999).

According to this meta-analysis, the effect of NO treatment on the shoot and root dry biomass accumulation was significant and more pronounced when the salt stress lasted for a more extended period (>21 days) (**Figures 4D**, **5D**). Several previous studies on maize (Keyster et al., 2012), rice (Habib et al., 2016), soybean (*Glycine max*) (Egbichi et al., 2014), and tomato (Wu et al., 2011a) demonstrated that exogenous NO confers salinity tolerance and improves shoot and root biomass during long-term salt exposure by lowering salt stress-induced oxidative stress and caspase-like activity through a pathway that restricts ROS formation *via* activating antioxidant machinery, stress-responsive gene expression, and molecular signaling. However, when plants were exposed to salinity for less than 20 days, NO treatment did not significantly increase plant biomass (**Figures 4D**, **5D**). These findings suggest that salinity tolerance mediated by NO is a slow but long-lasting process. As a result, it may be possible to mitigate salinity stress throughout the crop growing season effectively.

This meta-analysis showed that exogenous application of NO significantly improves shoot and root growth at both germination and seedling growth stages during salinity stress (**Figures 4E**, **5E**), suggesting that exogenous NO can be applied at both growth stages. NO promotes seed germination by breaking seed dormancy and modulating ABA signaling cascades under salinity conditions (Baudouin, 2011; Signorelli and Considine, 2018; Prakash et al., 2019). Moreover, NO alleviates salt-induced growth inhibition in plant seedlings by enhancing physiological and biochemical parameters (Ren et al., 2020), improving plant biomass accumulation. Studying the impacts of categorical factors on plant growth will aid us in identifying some potentially efficient plant–NO interactions that influence plant growth more strongly in the presence of salt. Interestingly, exogenous NO treatment promoted plant growth traits more effectively in dicot plants than in monocots under salinity stress conditions (**Figures 4F**, **5F**), as salt tolerance variation in response to NO is greater in dicots compared to monocot plants (Choudhary et al., 2022). Most of the research focused on NO-mediated salinity stress alleviation in graminoids and forbs type plants, and meta-analysis showed that the application of NO markedly increased shoot and root growth in these plants (**Figures 4G**, **5G**).

In this meta-analysis, most of the cases studied showed that, under salinity stress, the concentration of exogenous NO for improving shoot biomass ranges from 0.1 to 0.2 mM and for root biomass ranges from 0.05 to 0.2 mM (**Figures S1A, C** **)**. Furthermore, the meta-regression analysis revealed that the SMD for SDW or RDW did not change as NO concentration increased. As a result, we recommend a lower NO concentration (0.1 mM) as the optimal concentration for future experiments or field applications. Moreover, exogenous NO application can mitigate salinity stress up to 150 mM NaCl (**Figures S1B, D**).

### Does exogenous NO enhance photosynthesis in salt-induced plants?

Salinity stress severely affects the photosynthetic activity of plants by hampering chloroplast structure and function, reducing photosynthetic rate and interfering with stomatal conductance (Teixeira and Pereira, 2007; Chaves et al., 2009; Guidi et al., 2017; Sotiras et al., 2019; Landi et al., 2020). In NO-treated salt-stressed plants, all photosynthetic metrics included in this meta-analysis were significantly better than in non-NO-treated salt-stressed plants (**Figure 6**). In various methods, exogenous NO can assist plants in mitigating or reducing the negative impacts of salt stress on photosynthesis. As shown in our meta-analysis, NO improved plant osmotic adjustment and nutritional balance (**Figure 7**), allowing them to maintain a greater leaf area, higher chlorophyll content, stomatal conductance, and subcellular CO_2_ levels, all of which boosted CO_2_ assimilation rate and photosynthesis efficiency under normal and salt-stress conditions (Huang et al., 2016; Khoshbakht et al., 2018). Moreover, this meta-analysis showed that NO treatment significantly enhanced Ca^2+^ and Mg^2+^ in salt-stressed plants (**Figure 7**). Several studies have revealed that NO-treated plants have a more significant concentration of these cations, which could be linked to increased chlorophyll and carotenoid pigment production in NO-treated perennial ryegrass (*Lolium perenne*) (Wang et al., 2013), pepper (Shams et al., 2019), and maize (Kaya et al., 2015).

The appropriate presence of NO improves CO_2_ assimilation, transpiration, photosynthetic rate, and chlorophyll fluorescence characteristics, all of which improve photosynthetic functioning (Procházková et al., 2013; Alnusairi et al., 2021). Our results revealed that exogenous NO enhances transpiration, stomatal opening, and chlorophyll fluorescence in salt-affected plants (**Figure 6**). These results might be due to maintaining balanced K^+^ flux and inducing the expression of the plasma membrane H^+^-ATPase essential for an optimum K^+^/Na^+^ ion ratio to generate protection against salt stress by exogenous NO (Zhao et al., 2004); previously, similar findings were reported in tomato and barley (*Hordeum vulgare*) (Zhang et al., 2006; Wu et al., 2011a). Furthermore, it was demonstrated that NO application improves photosynthesis in salt-stressed mustard by enhancing RuBisCO enzyme activity and stomatal conductance (Fatma and Khan, 2014; Jahan et al., 2020). Nitric oxide alone or combined with sulfur promotes the synthesis of glutathione, assimilation of sulfur, and optimum production of NO and redox state, which represent the basis of NO-triggered defensive mechanisms of mustard plants (Fatma et al., 2016). The authors hypothesized that the application of NO increases GSH content, which plays a role in cellular redox homeostasis and regulation of stomatal movement. It was also demonstrated that GSH interacts with ABA to regulate stomatal movement (Misra et al., 2015).

### Does exogenous NO robust plants’ osmotic potential and nutrient balance under salt stress?

Our meta-analysis revealed that exogenous NO-enhanced essential minerals, e.g., K^+^, Ca^2+^, and Mg^2+^ uptake, and decreased toxic Na^+^ content in the salt-stressed plants (**Figure 7B**). Under salinity, plants invoke stress tolerance through ion homeostasis by increasing the K^+^/Na^+^ ratio, improving Ca^2+^/Mg^2+^ uptake, regulating ion uptake from roots, ion transport, and compartmentalization (Lu et al., 2014; Kolbert, 2016; Goyal et al., 2021). The Na^+^/H^+^ antiporter enzyme is involved in the elimination of cytosolic Na^+^ and the increase of Ca^2+^ and Mg^2+^ influx during this compartmentalization process (Chen et al., 2013; Goyal et al., 2021). Nitric oxide activates the H^+^-ATPase and H^+^-PPase enzymes in the vacuole, forcing the Na^+^/H^+^ ion exchange to detoxify the cell. Several studies have found that during Na^+^ compartmentalization, H^+^-ATPase activity increases. Under salt stress, NO mediates root K^+^/Na^+^ balance in maize (Zhang et al., 2006), sunflower (*Helianthus annus*) (David et al., 2010), and mangrove plant *Kandelia obovata* (Chen et al., 2013), *via* enhancing the expression of the AKT1-type K^+^ channel and Na^+^/H^+^ antiporter. It has also been reported that exogenous combined NO and H_2_S treatment increases salinity tolerance in barley by boosting H^+^-ATPase and Na^+^/H^+^ antiporter expression and K^+^ channel activity (Chen et al., 2015).

Osmotic stress caused by salinity can be alleviated by the buildup of osmolytes such as proline, soluble carbohydrates, and soluble proteins (Hayat et al., 2012a; Li et al., 2016). According to this meta-analysis (**Figure 7A**), the treatment of NO increases proline accumulation in plants but not soluble carbohydrates or soluble proteins under salt stress, suggesting that NO-mediated osmotic balance is mainly conferred by proline, and soluble sugar accumulation might not be stimulated by exogenous NO under salt stress. It has been previously reported that exogenous NO promotes the accumulation of osmotic components (e.g., proline and glycine betaine), which play an essential role in osmoregulation, membrane stability, cell water content maintenance, and stress mitigation in crop plants (Ahmad and Sharma, 2008; Ahanger et al., 2017). Exogenous NO has also been discovered to stimulate the P5CS1 gene, which codes for d1-pyrroline-5-carboxylate synthase, a critical enzyme involved in proline production in *Chlamydomonas reinhardtii* (Zhang et al., 2008; Rejeb et al., 2014). According to the review by Ahmad et al. (2016), NO-induced cell osmoregulation has been found in a variety of crops, including linseed (*Linum usitatissimum*), *Brassica*, chickpea, and mulberry (*Morus alba*).

Several investigations have demonstrated that exogenous NO treatment increases the level of soluble sugars and soluble proteins along with proline in salt-exposed plants through inducing osmotic homeostasis mechanisms, for example, salt-stress related sugars and proteins synthesis, CO_2_ assimilation, enzyme activities, and expression of specific genes (Gibson, 2005; Gupta and Kaur, 2005; Doganlar et al., 2010) in tomato (Wu et al., 2011a) and cotton (Dong et al., 2014). Our meta-analysis results showed no significant increase in soluble sugar and protein content (**Figure 7A**), which contradicted the previously described results. Further investigations are required to determine whether the exogenous NO triggers the possible pathways for increasing total soluble sugar and soluble protein levels during salinity stress.

### How does exogenous NO regulate plants’ antioxidant system and ROS scavenging during salt-induced stress?

Salt stress causes plants to accumulate many ROS, resulting in oxidative damage and a loss of membrane lipid and membrane integrity (Ahmad, 2010; Hayat et al., 2012a; AbdElgawad et al., 2016). This meta-analysis showed that NO supplementation detoxifies ROS generation and pacifies oxidative damage by significantly reducing MDA production and markedly boosting the antioxidants SOD, CAT, POX, APX, GR, DHAR, GSH, and ASC in salt-stressed plants (**Figure 8**). Farooq et al. (2009) and Qiao et al. (2014) previously described the mechanisms by which NO provides salt tolerance in plants by restoring redox equilibrium in salt-stressed plants by enhancing the activity of ROS scavenging enzymes such as CAT, SOD, APX, GR, and GPX. NO helps attenuate oxidative stress by upregulating both enzymatic and non-enzymatic antioxidants (Christou et al., 2014; Kong et al., 2016; da-Silva et al., 2018). The antioxidant enzymes in NO-treated plants were dramatically upregulated, and ROS-producing enzymes were downregulated, resulting in the rapid removal of excess accumulated free radicals and the stability of structural and functional elements of cellular membranes in agricultural plants like *Brassica oleracea* (Hernandez et al., 2010), maize (Carrasco-Ríos and Pinto, 2014), pepper (Shams et al., 2019), and wheat (Alnusairi et al., 2021).

NO can operate as a signaling molecule or ROS scavenger by modulating or boosting the activities of antioxidant enzymes under sustained stress circumstances (Hao et al., 2008; Xu et al., 2010; Fan and Liu, 2012). The expression of representative *SOD*, *CAT*, and *APX* genes examined was upregulated by exogenous NO treatment in salt-stressed plants like soybean (Egbichi et al., 2014), chickpea (Ahmad et al., 2016), tomato (Aydin et al., 2014), *Limonium sinense* (Zhang et al., 2014), *Lotus japonicus* (Rubio Luna et al., 2009) and rice (Shafi et al., 2015). Similarly, this meta-analysis indicates that upregulation of *CAT*, *SOD*, *APX*, *GR*, *GPX*, and *DHAR* genes might enhance the activities of the SOD, CAT, APX, and DHAR enzymes; as a result, the cells are better protected against oxidative damage caused by high salinity (Lu et al., 2007; Yamane et al., 2010; Hu et al., 2012). Therefore, NO stimulates the expression of antioxidant enzyme-related biosynthetic genes, resulting in the buildup of antioxidant enzymes and hence greater stress tolerance in plants. NO suppress oxidative damage and avert cell membrane injury and proton extrusion through upregulated H^+^-ATPase activity in stressed plants (Kharbech et al., 2020). Reports have suggested that NO-signaling helps to regulate this cycle *via S*-nitrosylation. Under salt-induced stress, the activities of four vital enzymes of the ASC–GSH cycle, namely, APX, DHAR, and GR, are hampered (Rahman et al., 2016). Furthermore, NO and sulfur combine to increase APX activity, allowing it to efficiently detoxify H_2_O_2_ and O_2_•, resulting in a robust antioxidative defense against salinity (Fatma and Khan, 2014; Fatma et al., 2016).

### Recommendations and research gaps

The current systematic review emphasizes the role of NO-mediated plant salinity tolerance and exogenous NO application in alleviating plant salinity stress. This meta-analysis also highlights various aspects that require further investigation to gain a thorough and robust understanding of NO-conferred salinity stress tolerance in plants. Based on this meta-analysis, we enclose the following recommendations or research gaps:

• The majority of research has concentrated on the effects of NO on plant physiology by utilizing seed priming and foliar application under saline conditions. However, there has been less attention dedicated to determining the effects of NO in root medium-based approaches. Before advocating for root medium-based treatments, we need further evidence on the variability and durability of NO-induced salinity tolerance.
• From a mechanistic standpoint, most articles measured osmolyte content in NO-treated and non-NO-treated plants, but the underlying mechanisms of this response at the molecular level need to be investigated further.
• Plant lipid metabolism is altered during salt stress, which is linked to changes in membrane integrity, constitution, and activity (Parihar et al., 2015). Although lipid peroxidation has been examined in salt-stressed NO-treated plants, overall changes in lipid metabolism in response to exogenous NO have gained less priority.
• Very few studies have described the effects of exogenous NO on the nutritional aspects of crops. We have yet to explore whether the contents of bioactive nutritional components are improved or decreased by NO under salt stress.
• Salinity stress limits plant growth and biomass building by disrupting photosynthetic pigments, osmotic equilibrium, enzyme activity, and ionic homeostasis. Exogenously applied NO reliably mitigates the impact of salt stress on plants by regulating their physiological functions and oxidative tolerance. NO-mediated salinity stress tolerance in plants has far-reaching ecological and agro-economic implications. Improved salinity tolerance may ensure higher crop yields in some agronomic fields. Continued research on exogenous NO application could lead to discovering the fundamental processes of plant-NO interactions, which appear to be well-founded.
• Many previous studies and our meta-analysis proved that exogenous NO application significantly improves the growth and yield of plants. However, there was no conclusive data and findings on the economic feasibility of NO-mediated crop cultivation in the saline-pone agronomic fields. A further investigation is recommended to study the impact level of exogenous NO on crop yield performance following the economic profitability and cost-effectiveness of farmers.
• NO treatment effect on C3 plants has been extensively described, whereas only a few investigations on C4 plants have been conducted under salt stress. More studies on exogenous NO-mediated salinity tolerance in C4 plants are needed to get a definitive conclusion.
• Although many studies on exogenous NO-induced salinity tolerance have been reported at germination stage in field levels, a limited study was conducted at the reproductive stage. Therefore, the ameliorative role of NO on plant growth and yields at the reproductive stage in field conditions needs to be investigated further.
• Many of these experiments were undertaken in greenhouses or under controlled growth chamber conditions, with limited investigations conducted at the field level. Further research on the variability and stability of NO-induced salinity tolerance is required before farm-level recommendations.
• Continuous NO application in root did not positively affect but rather had adverse effects. Thus, future studies should avoid continuous NO application for mitigating salinity stress.
• Typically, studies are conducted on a single method. Further research should be conducted in which NO will be used in various methods to provide a comparative conclusion. Furthermore, different methods should be used throughout the plant life cycle in a single study. For example, studies should be conducted in which seed priming with NO will be applied at the seedling stage and foliar pretreatment at the vegetative or reproductive stage.

## Conclusion

The present meta-analysis of 62 peer-reviewed articles demonstrated the NO-mediated morpho-physiological and biochemical response and stress tolerance mechanisms in plants under salinity conditions. In particular, we determined the effects of different sub-categories in NO-treated and salt-stressed plants. Exogenous application of NO considerably influences biomass accumulation, growth, and yield in both salt-stressed and non-stressed plants. Our meta-analysis reveals that exogenous NO application is more effective when the salt-stressed plants are grown in growth chambers and at germination and seedling growth stages under prolonged salinity stress. The number of field-level reproductive stage experiments is minimal, and future research should concentrate on this topic. Among the NO application methods, seed priming and foliar pre-treatment are the most efficient when NO is applied for a one-time or regular interval. Furthermore, the optimum NO concentration ranges from 0.1 to 0.2 mM, which alleviates salinity stress up to 150 mM. Interestingly, exogenous NO treatment boosts plant growth most efficiently in dicots. This meta-analysis shows exogenous NO strongly confers plant salinity stress tolerance by maintaining physiological and biochemical processes such as osmotic and nutritional balance, and photosynthetic and antioxidant activity. Further investigations are recommended to study the roles of lipid metabolism, nutritional content changes of grain crops, responses of C4 plants, and field-level crop yield improvements under salinity stress in response to exogenous NO application.

## Data availability statement

The original contributions presented in the study are included in the article/**Supplementary Materials**. Further inquiries can be directed to the corresponding authors.

## Author contributions

MTUA developed the concept, performed the database search, partially collected the data, analyzed and interpreted the results, and wrote the draft. IJ, ZHS, AAMS, SHT, and IH extracted the data. KMGD analyzed the data and produced graphs. MH and IJ wrote the draft. XW, FZ, JC, RI, IA, and MM revised the manuscript. MTUA, AES, and YM revised the manuscript and supervised the project. All authors approved the final version of the manuscript.

## Conflict of interest

The authors declare that the research was conducted in the absence of any commercial or financial relationships that could be construed as a potential conflict of interest.

## Publisher’s note

All claims expressed in this article are solely those of the authors and do not necessarily represent those of their affiliated organizations, or those of the publisher, the editors and the reviewers. Any product that may be evaluated in this article, or claim that may be made by its manufacturer, is not guaranteed or endorsed by the publisher.

## Supplementary material

Supplementary Figure 1 Effects of different concentrations of NO and salinity on SDW **(A, B)** and RDW **(C, D)** of different plant species.

Supplementary Table 1 List of published research articles used for data extraction in this meta-analysis.

Supplementary Table 2 Comparative effects of different salinity levels on shoot and root biomass production in NO-treated plants.

Supplementary Table 3 Heterogeneity statistics for the growth-related traits summary effect sizes under non-saline and saline stress conditions.
