# Supplementary material for: Exogenous nitric oxide promotes salinity tolerance in plants: A meta-analysis
Source: Front Plant Sci. 2022 Nov 7;13:957735. doi: 10.3389/fpls.2022.957735 (PMC9676926; doi:10.3389/fpls.2022.957735)
Supplement: Supplementary Figure 1 — Effects of different concentrations of NO and salinity on SDW (A, B) and RDW (C, D) of different plant species. [file DataSheet_1.docx]

**Supplementary files**

**
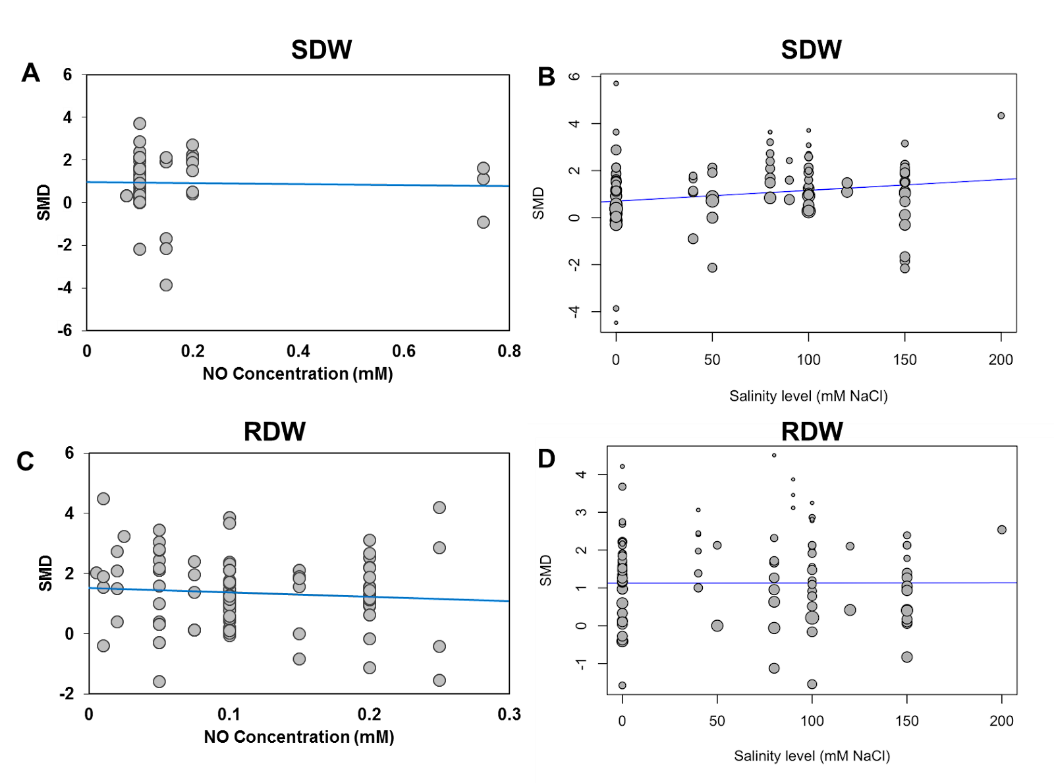
**

**Supplementary Figure S1:** Effects of different concentrations of NO and salinity on SDW (A, B) and RDW (C, D) of different plant species.

**Supplementary Table 1:** List of published research articles used for data extraction in this meta-analysis.

1. Adamu TA, Mun BG, Lee SU, Hussain A, Yun BW. Exogenously applied nitric oxide enhances salt tolerance in rice (Oryza sativa L.) at seedling stage. Agronomy. 2018 Dec;8(12):276.
2. Ahanger MA, Aziz U, Alsahli AA, Alyemeni MN, Ahmad P. Influence of exogenous salicylic acid and nitric oxide on growth, photosynthesis, and ascorbate-glutathione cycle in salt stressed Vigna angularis. Biomolecules. 2020 Jan;10(1):42.
3. Ahmad P, Abdel Latef AA, Hashem A, Abd_Allah EF, Gucel S, Tran LS. Nitric oxide mitigates salt stress by regulating levels of osmolytes and antioxidant enzymes in chickpea. Frontiers in Plant Science. 2016 Mar 31;7:347.
4. Akram NA, Hafeez N, Farid-ul-Haq M, Ahmad A, Sadiq M, Ashraf M. Foliage application and seed priming with nitric oxide causes mitigation of salinity-induced metabolic adversaries in broccoli (Brassica oleracea L.) plants. Acta Physiologiae Plantarum. 2020 Oct;42(10):1-9.
5. Ali Q, Daud MK, Haider MZ, Ali S, Rizwan M, Aslam N, Noman A, Iqbal N, Shahzad F, Deeba F, Ali I. Seed priming by sodium nitroprusside improves salt tolerance in wheat (Triticum aestivum L.) by enhancing physiological and biochemical parameters. Plant physiology and biochemistry. 2017 Oct 1;119:50-8.
6. Aras S, Keles H, Eşitken A. SNP mitigates malignant salt effects on apple plants. Erwerbs-Obstbau. 2020 Mar;62(1):107-15.
7. Babri-Bonab R, Saadatmand S, Nazemiyeh H, Iran-Bakhsh A. The effect of different concentrations of exogenous nitric oxide on several physiological and biochemical parameters in NaCl-stressed coriander (Coriandrum sativum L.). Iranian Journal of Plant Physiology. 2018 Aug 1;8(4):2517-24.
8. Bai X, Yang L, Yang Y, Ahmad P, Yang Y, Hu X. Deciphering the protective role of nitric oxide against salt stress at the physiological and proteomic levels in maize. Journal of proteome research. 2011 Oct 7;10(10):4349-64.
9. Boldizsár Á, Simon-Sarkadi L, Szirtes K, Soltész A, Szalai G, Keyster M, Ludidi N, Galiba G, Kocsy G. Nitric oxide affects salt-induced changes in free amino acid levels in maize. Journal of plant physiology. 2013 Jul 15;170(11):1020-7.
10. Campos FV, Oliveira JA, Pereira MG, Farnese FS. Nitric oxide and phytohormone interactions in the response of Lactuca sativa to salinity stress. Planta. 2019 Nov;250(5):1475-89.
11. Chen J, Xiao Q, Wang C, Wang WH, Wu FH, He BY, Zhu Z, Ru QM, Zhang LL, Zheng HL. Nitric oxide alleviates oxidative stress caused by salt in leaves of a mangrove species, Aegiceras corniculatum. Aquatic botany. 2014 Jul 1;117:41-7.
12. Christou A, Manganaris GA, Fotopoulos V. Systemic mitigation of salt stress by hydrogen peroxide and sodium nitroprusside in strawberry plants via transcriptional regulation of enzymatic and non-enzymatic antioxidants. Environmental and Experimental Botany. 2014 Nov 1;107:46-54.
13. Dinler BS, Antoniou C, Fotopoulos V. Interplay between GST and nitric oxide in the early response of soybean (Glycine max L.) plants to salinity stress. Journal of plant physiology. 2014 Nov 15;171(18):1740-7.
14. Dong YJ, Jinc SS, Liu S, Xu LL, Kong J. Effects of exogenous nitric oxide on growth of cotton seedlings under NaCl stress. Journal of soil science and plant nutrition. 2014 Mar;14(1):1-3.
15. Dong YJ, Wang ZL, Zhang JW, Liu S, He ZL, He MR. Interaction effects of nitric oxideand salicylic acid in alleviating salt stress of Gossypium hirsutum L. Journal of soil science and plant nutrition. 2015 Sep;15(3):561-73.
16. Dong YJ, Zhang Q, Dai XL, He MR. Effects of potassium chloride and nitric oxide on growth and physiological characteristics of winter wheat under salt stress. Biologia plantarum. 2020 Mar 19;64:258-65.
17. Du ST, Liu Y, Zhang P, Liu HJ, Zhang XQ, Zhang RR. Atmospheric application of trace amounts of nitric oxide enhances tolerance to salt stress and improves nutritional quality in spinach (Spinacia oleracea L.). Food Chemistry. 2015 Apr 15;173:905-11.
18. Egbichi I, Keyster M, Ludidi N. Effect of exogenous application of nitric oxide on salt stress responses of soybean. South African Journal of Botany. 2014 Jan 1;90:131-6.
19. Fan H, Guo S, Jiao Y, Zhang R, Li J. Effects of exogenous nitric oxide on growth, active oxygen species metabolism, and photosynthetic characteristics in cucumber seedlings under NaCl stress. Frontiers of Agriculture in China. 2007 Jul 1;1(3):308-14.
20. Fan HF, Du CX, Ding L, Xu YL. Effects of nitric oxide on the germination of cucumber seeds and antioxidant enzymes under salinity stress. Acta physiologiae plantarum. 2013 Sep 1;35(9):2707-19.
21. Fan HF, Du CX, Guo SR. Effect of nitric oxide on proline metabolism in cucumber seedlings under salinity stress. Journal of the American Society for Horticultural Science. 2012 May 1;137(3):127-33.
22. Fan HF, Du CX, Guo SR. Nitric oxide enhances salt tolerance in cucumber seedlings by regulating free polyamine content. Environmental and Experimental Botany. 2013 Feb 1;86:52-9.
23. Fatma M, Masood A, Per TS, Khan NA. Nitric oxide alleviates salt stress inhibited photosynthetic performance by interacting with sulfur assimilation in mustard. Frontiers in plant science. 2016 Apr 25;7:521.
24. Gohari G, Alavi Z, Esfandiari E, Panahirad S, Hajihoseinlou S, Fotopoulos V. Interaction between hydrogen peroxide and sodium nitroprusside following chemical priming of Ocimum basilicum L. against salt stress. Physiologia plantarum. 2020 Feb;168(2):361-73.
25. Habib N, Akram MS, Javed MT, Azeem M, Ali Q, Shaheen HL, Ashraf M. Nitric oxide regulated improvement in growth and yield of rice plants grown under salinity stress: antioxidant defense system. Appl Ecol Environ Res. 2016 Jan 1;14(5):91-105.
26. Hasanuzzaman M, Hossain MA, Fujita M. Nitric oxide modulates antioxidant defense and the methylglyoxal detoxification system and reduces salinity-induced damage of wheat seedlings. Plant Biotechnology Reports. 2011 Oct;5(4):353-65.
27. Hayat S, Yadav S, Wani AS, Irfan M, Alyemini MN, Ahmad A. Impact of sodium nitroprusside on nitrate reductase, proline content, and antioxidant system in tomato under salinity stress. Horticulture, Environment, and Biotechnology. 2012 Oct 1;53(5):362-7.
28. Jamali B, Eshghi S, Tafazoli E. Mineral composition of ‘Selva’strawberry as affected by time of application of nitric oxide under saline conditions. Horticulture, Environment, and Biotechnology. 2015 Jun;56(3):273-9.
29. Karami A, Sepehri A. Nano titanium dioxide and nitric oxide alleviate salt induced changes in seedling growth, physiological and photosynthesis attributes of barley. Zemdirbyste-Agriculture. 2018 Apr 1;105(2).
30. Kaur H, Bhatla SC. Melatonin and nitric oxide modulate glutathione content and glutathione reductase activity in sunflower seedling cotyledons accompanying salt stress. Nitric Oxide. 2016 Sep 30;59:42-53.
31. Keyster M, Klein A, Ludidi N. Caspase-like enzymatic activity and the ascorbate-glutathione cycle participate in salt stress tolerance of maize conferred by exogenously applied nitric oxide. Plant signaling & behavior. 2012 Mar 1;7(3):349-60.
32. Khan MN, Siddiqui MH, Mohammad F, Naeem M. Interactive role of nitric oxide and calcium chloride in enhancing tolerance to salt stress. Nitric Oxide. 2012 Dec 1;27(4):210-8.
33. Klein A, Hüsselmann L, Keyster M, Ludidi N. Exogenous nitric oxide limits salt-induced oxidative damage in maize by altering superoxide dismutase activity. South African journal of botany. 2018 Mar 1;115:44-9.
34. Klein A, Hüsselmann L, Keyster M, Ludidi N. Exogenous nitric oxide limits salt-induced oxidative damage in maize by altering superoxide dismutase activity. South African journal of botany. 2018 Mar 1;115:44-9.
35. Li QY, Niu HB, Yin J, Wang MB, Shao HB, Deng DZ, Chen XX, Ren JP, Li YC. Protective role of exogenous nitric oxide against oxidative-stress induced by salt stress in barley (Hordeum vulgare). Colloids and Surfaces B: Biointerfaces. 2008 Sep 1;65(2):220-5.
36. Liu A, Fan J, Gitau MM, Chen L, Fu J. Nitric oxide involvement in bermudagrass response to salt stress. Journal of the American Society for Horticultural Science. 2016 Sep 1;141(5):425-33.
37. Liu S, Dong Y, Xu L, Kong J. Effects of foliar applications of nitric oxide and salicylic acid on salt-induced changes in photosynthesis and antioxidative metabolism of cotton seedlings. Plant Growth Regulation. 2014 May 1;73(1):67-78.
38. Liu S, Dong YJ, Xu LL, Kong J, Bai XY. Roles of exogenous nitric oxide in regulating ionic equilibrium and moderating oxidative stress in cotton seedlings during salt stress. Journal of soil science and plant nutrition. 2013 Dec;13(4):929-41.
39. Nalousi AM, Ahmadiyan S, Hatamzadeh A, Ghasemnezhad M. Protective role of exogenous nitric oxide against oxidative stress induced by salt stress in bell-pepper (Capsicum annum L.). American Eurasian Journal of Agricultural & Environmental Sciences. 2012;12:1085-90.
40. Nejadalimoradi HA, Nasibi FA, Kalantari KM, Zanganeh RO. Effect of seed priming with L-arginine and sodium nitroprusside on some physiological parameters and antioxidant enzymes of sunflower plants exposed to salt stress. Agric Commun. 2014;2(1):23-30.
41. Noman HM and Shahbaz M. Effect of exogenously applied nitric oxide on some key physiological attributes of rice (Oryza sativa L.) plants under salt stress. Pak. J. Bot. 2013;45(5):1563-9.
42. Ren Y, Wang W, He J, Zhang L, Wei Y, Yang M. Nitric oxide alleviates salt stress in seed germination and early seedling growth of pakchoi (Brassica chinensis L.) by enhancing physiological and biochemical parameters. Ecotoxicology and environmental safety. 2020 Jan 15;187:109785.
43. Salahuddin M, Nawaz F, Shahbaz M, Naeem M, Zulfiqar B, Shabbir RN, Hussain RA. Effect of exogenous nitric oxide (NO) supply on germination and seedling growth of mungbean (cv. Nm-54) under salinity stress. Legume Research-An International Journal. 2017;40(5):846-52.
44. Shams M, Ekinci M, Ors S, Turan M, Agar G, Kul R, Yildirim E. Nitric oxide mitigates salt stress effects of pepper seedlings by altering nutrient uptake, enzyme activity and osmolyte accumulation. Physiology and Molecular Biology of Plants. 2019 Sep 1;25(5):1149-61.
45. Shen ZJ, Chen J, Ghoto K, Hu WJ, Gao GF, Luo MR, Li Z, Simon M, Zhu XY, Zheng HL. Proteomic analysis on mangrove plant Avicennia marina leaves reveals nitric oxide enhances the salt tolerance by up-regulating photosynthetic and energy metabolic protein expression. Tree Physiology. 2018 Nov;38(11):1605-22.
46. Sheokand S, Bhankar V, Sawhney V. Ameliorative effect of exogenous nitric oxide on oxidative metabolism in NaCl treated chickpea plants. Brazilian Journal of Plant Physiology. 2010;22(2):81-90.
47. Sheokand S, Kumari A, Sawhney V. Effect of nitric oxide and putrescine on antioxidative responses under NaCl stress in chickpea plants. Physiology and Molecular Biology of Plants. 2008 Oct 1;14(4):355-62.
48. Shi Q, Ding F, Wang X, Wei M. Exogenous nitric oxide protect cucumber roots against oxidative stress induced by salt stress. Plant Physiology and Biochemistry. 2007 Aug 1;45(8):542-50.
49. Shi Q, Ding F, Wang X, Wei M. Exogenous nitric oxide protect cucumber roots against oxidative stress induced by salt stress. Plant Physiology and Biochemistry. 2007 Aug 1;45(8):542-50.
50. Siddiqui MH, Alamri SA, Al-Khaishany MY, Al-Qutami MA, Ali HM, Hala AR, Kalaji HM. Exogenous application of nitric oxide and spermidine reduces the negative effects of salt stress on tomato. Horticulture, Environment, and Biotechnology. 2017 Dec 1;58(6):537-47.
51. Simaei M, Khavari-Nejad RA, Bernard F. Exogenous application of salicylic acid and nitric oxide on the ionic contents and enzymatic activities in NaCl-stressed soybean plants.
52. Simaei M, Khavarinejad RA, Saadatmand S, Bernard F, Fahimi H. Interactive effects of salicylic acid and nitric oxide on soybean plants under NaCl salinity. Russian Journal of Plant Physiology. 2011 Sep 1;58(5):783.
53. Tanou G, Molassiotis A, Diamantidis G. Hydrogen peroxide-and nitric oxide-induced systemic antioxidant prime-like activity under NaCl-stress and stress-free conditions in citrus plants. Journal of plant physiology. 2009 Nov 15;166(17):1904-13.
54. Tian X, He M, Wang Z, Zhang J, Song Y, He Z, Dong Y. Application of nitric oxide and calcium nitrate enhances tolerance of wheat seedlings to salt stress. Plant Growth Regulation. 2015 Dec 1;77(3):343-56.
55. Wu X, Zhu W, Zhang H, Ding H, Zhang HJ. Exogenous nitric oxide protects against salt-induced oxidative stress in the leaves from two genotypes of tomato (Lycopersicom esculentum Mill.). Acta physiologiae plantarum. 2011 Jul 1;33(4):1199-209.
56. Wu XX, Zhu XH, Chen JL, Yang SJ, Ding HD, Zha DS. Nitric oxide alleviates adverse salt-induced effects by improving the photosynthetic performance and increasing the anti-oxidant capacity of eggplant (Solanum melongena L.). The Journal of Horticultural Science and Biotechnology. 2013 Jan 1;88(3):352-60.Yadu S, Dewangan TL, Chandrakar V, Keshavkant S. Imperative roles of salicylic acid and nitric oxide in improving salinity tolerance in Pisum sativum L. Physiology and molecular biology of plants. 2017 Jan 1;23(1):43-58.
57. Yurekli F, Kirecci OA, Celik I. The effects of nitric oxide on some antioxidant enzyme activities under salt stress in sunflower plants. Acta Scientiarum Polonorum. Hortorum Cultus. 2019 Jan 1;18(5).
58. Yurekli F, Kirecci OA. The relationship between nitric oxide and plant hormones in SNP administrated sunflower plants under salt stress condition. Acta Sci. Pol. Hortorum Cultus. 2016 Jan 1;15(6):177-91.
59. Zavareh M. Protective effect of exogenous nitric oxide on alleviation of oxidative damage induced by high salinity in rice (Oryza sativa L.) seedlings. Iran Agricultural Research. 2015 Jun 30;34(1):63-70.
60. Zeng CL, Liu L, Wang BR, Wu XM, Zhou Y. Physiological effects of exogenous nitric oxide on Brassica juncea seedlings under NaCl stress. Biologia Plantarum. 2011 Jun 1;55(2):345-8.
61. Zhang Y, Wang L, Liu Y, Zhang Q, Wei Q, Zhang W. Nitric oxide enhances salt tolerance in maize seedlings through increasing activities of proton-pump and Na+/H+ antiport in the tonoplast. Planta. 2006 Aug;224(3):545-55.
62. Zheng C, Jiang D, Liu F, Dai T, Liu W, Jing Q, Cao W. Exogenous nitric oxide improves seed germination in wheat against mitochondrial oxidative damage induced by high salinity. Environmental and Experimental Botany. 2009 Nov 1;67(1):222-7.

**Supplementary Table 2:** Comparative effects of different salinity levels on shoot and root biomass production in NO-treated plants

| **Parameters** | **Salinity level** | **SMD** | **L95%CI** | **U95%CI** | **p-value** | **Welch t-test**  *p*=0.661 |
| --- | --- | --- | --- | --- | --- | --- |
| **SDW** | Low salinity | 0.6818 | 0.1430 | 1.2207 | 0.0150 | *p*=0.697 |
|  | Moderate salinity | 0.9566 | 0.6227 | 1.2905 | <0.0001 | *p*=0.661 |
|  | High salinity | 0.6424 | -0.1474 | 1.4322 | 0.1058 | *p*=0.386 |
| **RDW** | Low salinity | 0.7973 | 0.2274 | 1.3671 | 0.0082 | *p*=0.932 |
|  | Moderate salinity | 0.6001 | 0.0737 | 1.1264 | 0.0268 | *p*=0.418 |
|  | High salinity | 0.7541 | 0.2563 | 1.2519 | 0.0048 |  |

**Supplementary Table 3:** Heterogeneity statistics for the growth-related traits summary effect sizes under non-saline and saline stress conditions

| **Para-meters** | **Salinity level** | ***tau^2^*** | ***tau*** | ***I^2^*** | **H** | **Q; d.f.**  ***p* value** |
| --- | --- | --- | --- | --- | --- | --- |
| **RL** | **Non-saline** | 0.8382  [0.2671; 13.5334] | 0.9155  [0.5168; 3.6788] | 51.1%  [17.2%; 71.1%] | 1.43  [1.10; 1.86] | 36.80; 18  0.0056 |
|  | **Saline** | 0.0400  [0.0836; 7.4025] | 0.1999  [0.2892; 2.7208] | 42.1%  [7.7%; 63.7%] | 1.31  [1.04; 1.66] | 43.19; 25  0.0133 |
| **SL** | **Non-saline** | <0.0001  [0.0000; 1.0603] | 0.0016  [0.0000; 1.0297] | 0.0%  [0.0%; 37.1%] | 1.00  [1.00; 1.26] | 28.49; 30  0.5445 |
|  | **Saline** | <0.0001  [0.0000; 1.4527] | 0.0008  [0.0000; 1.2053] | 21.0%  [0.0%; 42.4%] | 1.13  [1.00; 1.32] | 81.00; 64  0.0743 |
| **SDW** | **Non-saline** | <0.0001  [0.0132; 2.0166] | 0.0019  [0.1147; 1.4201] | 31.2%  [1.9%; 51.7%] | 1.21  [1.01; 1.44] | 69.75; 48  0.0218 |
|  | **Saline** | 0.6198  [0.3832; 1.8339] | 0.7872  [0.6190; 1.3542] | 42.0%  [25.0%; 55.2%] | 1.31  [1.15; 1.49] | 148.34; 86  <0.0001 |
| **RDW** | **Non-saline** | 0.2915  [0.1037; 1.6143] | 0.5399  [0.3220; 1.2706] | 36.0%  [10.1%; 54.4%] | 1.25  [1.05; 1.48] | 79.67; 51  0.0063 |
|  | **Saline** | 0.3631  [0.1851; 1.6035] | 0.6026  [0.4303; 1.2663] | 35.4%  [14.6%; 51.1%] | 1.24  [1.08; 1.43] | 119.12; 77  0.0015 |
| **Yield** | **Non-saline** | 0  [<0.00; <0.00] | 0  [<0.000; <0.000] | 0.0%  [0.0%; 0.0%] | 1.00  [1.00; 1.00] | 3.82; 24  1.0000 |
|  | **Saline** | < 0.0001  [0.3851; 5.4415] | 0.0027  [0.6205; 2.3327] | 45.9%  [19.1%; 63.9%] | 1.36  [1.11; 1.66] | 61.04; 33  0.0021 |

Q, total heterogeneity; *p*, significance of Q heterogeneity; *I^2^*, percentage of heterogeneity due to true variation in effect sizes.
